# Unraveling biochemical spatial patterns: Machine learning approaches to the inverse problem of stationary Turing patterns

**DOI:** 10.1016/j.isci.2024.109822

**Published:** 2024-04-29

**Authors:** Antonio Matas-Gil, Robert G. Endres

**Affiliations:** 1 Department of Life Sciences & Centre for Integrative Systems Biology and Bioinformatics, Imperial College London, London SW7 2BU, UK

**Keywords:** Biochemistry, Machine learning

## Abstract

The diffusion-driven Turing instability is a potential mechanism for spatial pattern formation in numerous biological and chemical systems. However, engineering these patterns and demonstrating that they are produced by this mechanism is challenging. To address this, we aim to solve the inverse problem in artificial and experimental Turing patterns. This task is challenging since patterns are often corrupted by noise and slight changes in initial conditions can lead to different patterns. We used both least squares to explore the problem and physics-informed neural networks to build a noise-robust method. We elucidate the functionality of our network in scenarios mimicking biological noise levels and showcase its application using an experimentally obtained chemical pattern. The findings reveal the significant promise of machine learning in steering the creation of synthetic patterns in bioengineering, thereby advancing our grasp of morphological intricacies within biological systems while acknowledging existing limitations.

## Highlights

• Turing patterns are relevant to biology but sensitive to noise and initial conditions
• Solving the inverse problem is crucial for the engineering synthetic Turing patterns
• Least squares excel in noise-free conditions with minimal amount of data
• Modified physics-informed neural network can deal with experimental noise levels

## Graphical abstract

**Figure:**
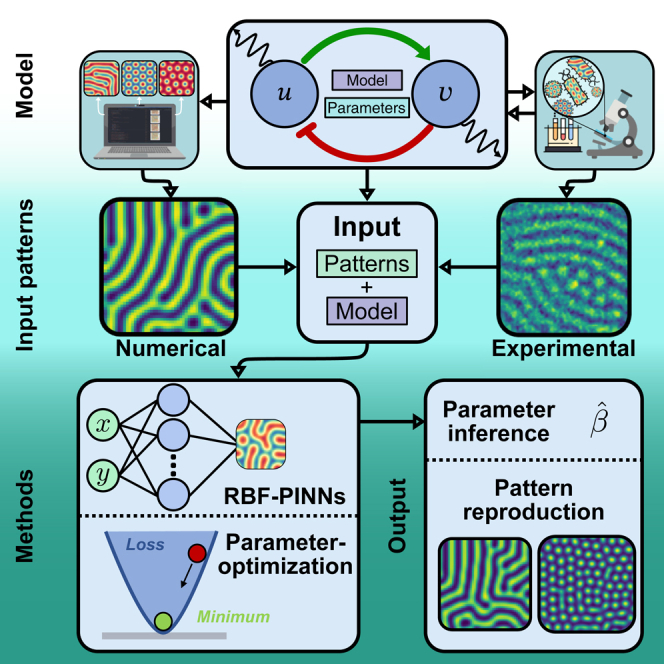

## Introduction

Spatial patterns are prevalent in biological systems, including gene expression in microbial communities, developing embryos, as well as skin and fur patterns in adult animals. A leading mechanism for pattern formation is the diffusion-driven instability in reaction-diffusion models, as proposed by Alan Turing^1^ and extended by others.^2^^,^^3^^,^^4^^,^^5^^,^^6^ These models typically describe interacting and diffusing activator and inhibitor chemical species through sets of coupled partial differential equations (PDEs), although other methods exist, such as cellular automata.^7^ While Turing patterns are observed in chemical systems,^8^^,^^9^^,^^10^^,^^11^ it has been challenging to conclusively demonstrate this mechanism in developmental systems such as digit formation,^12^^,^^13^ zebrafish skin pigment patterning,^14^ and hair spacing in mice^15^ (see also recent reviews^16^^,^^17^). Additionally, building Turing patterns from the bottom up with synthetic circuits has proven difficult.^18^^,^^19^ Identified issues are that Turing models appear abstract and unrealistic compared to actual biological regulatory pathways, are highly sensitive to changes in model parameters,^20^ and experimental control over parameter tuning is limited. Even if patterns can be produced experimentally, the challenge remains of linking them to the corresponding parameters of a candidate model to support them as an actual Turing pattern rather than an experimental artifact caused, e.g., by chemical gradients, boundary conditions, or prepatterns. Further complications arise from the relatively limited amount of data available in developmental systems, which are often corrupted by measurement and imaging noise, as well as the strong pattern variability observed in microbial systems. A way to address these issues is the development of tools to robustly estimate parameters from given patterns and candidate models, which is usually referred to as the inverse problem. In particular, with patterns engineered by synthetic biology,^21^ model selection is less of an issue, allowing one to focus on parameter estimation.

To explore the issues that arise in an experimental setting, the inverse problem can initially be approached using artificial data from numerical simulations instead of actual experimental data. Once a sufficiently noise-robust method is found, one can test it with experimental data. Numerical simulations involve generating random initial conditions and evolving the reaction-diffusion model in space and time, allowing for a systematic study of the effects of data size and noise. The focus of this paper is on steady-state patterns; hence, simulations are evolved until the dynamics converge. This means dynamics of the patterns are not explored. Nevertheless, solving the inverse problem remains a challenging task even with this simplification, as the same model parameters can produce different patterns, resulting in a many-to-one inversion problem.^22^ This occurs because patterns are highly sensitive to initial conditions, and even slight alterations can cause noticeable changes in the final pattern obtained. These changes do not generally affect the type of pattern obtained, such as spots or stripes, but can alter the location and shape of the pattern elements. (Exceptions may exist if more than one type of pattern is stable in certain regions of parameter space.^23^) Due to the difficulty posed by solving the inverse problem, focus has been on advanced approaches, including Bayesian inference and other statistical tools^24^^,^^25^ and different machine-learning techniques such as support vector machines, Kernel-based methods, and neural networks,^26^ as well as optimal control theory.^27^ However, despite some success, these approaches suffer from requiring thousands of training images,^25^ sensitivity to noise in the patterns,^26^ ad hoc cost or loss functions for quantifying the quality of fit,^24^ or the requirement to fix some parameters and have knowledge of initial conditions and the pattern evolution.^27^

As the inverse Turing problem remains largely unresolved, there is a need to develop new robust approaches, particularly when dealing with small and noisy data. Although conceptually straightforward, the least squares (LS) approach has not been extensively explored in this context since previous research considered it an ill-posed problem due to the many-to-one mapping from patterns to parameters.^24^ Generalized LS methods, however, found use in nonlinear regression problems with spatially correlated errors.^28^ Nevertheless, this method appears naturally fitting to the inverse Turing problem, since by discretizing the patterns and the Laplacians, the model equations can be written as an overdetermined system of equations dependent on the patterns, with the parameters being the variables. The best parameter combination is then trivially given by LS.^29^ In contrast, deep learning methods, such as neural networks, are usually more reliable when it comes to noise, even though they are computationally expensive to train. A key property that makes neural networks a promising tool for approaching this problem is the universal approximation theorem, which states that given enough parameters, neural networks are capable of approximating any continuous function, including spatial patterns.^30^^,^^31^ Specifically, physics-informed neural networks (PINNs) incorporate physical constraints, such as the model equations that should hold for the data, thereby helping to regularize the training^32^ and making PINNs more intuitive than black-box neural networks. An added benefit of PINNs is that they do not require large amounts of data,^33^ since they make use of physical laws to enforce additional constraints on the problem. To achieve this, model parameters are regarded as parameters of the neural network and simultaneously optimized, resulting in the computational cost of the whole method being of the same order as the standalone function approximation. This makes PINNs superb candidates for solving the inverse problem in Turing patterns.

Here, we explore basic LS applied to the PDE residuals and advanced PINNs to address the inverse problem - given stationary Turing patterns and candidate models, we aim to recover the model parameters. Using our first approach, we find that LS is computationally inexpensive, but that it requires mostly exact (albeit little) data and hence does not allow significant noise or using a similar pattern produced from a different model. Applying this methodology to small regions of patterns still allows us to recover the parameters, and we identify the minimum number of necessary pixels. Our second approach uses PINNs with a radial basis function (RBF) architecture to approximate the patterns, referred to as RBF-PINNs. We found this architecture to perform better than traditional PINNs and be more interpretable, thereby aligning with the white-box neural networks idea of fully understanding the mapping between inputs and the outcomes, in contrast to black-box networks where this mapping is disregarded. This method remedies many of the issues from the first method at a two-order of magnitude higher computational costs, allowing us to obtain accurate results up to 10–20% relative noise given a single snapshot of a Turing pattern, even in an experimental setting with chemical Turing patterns. Hence, our RBF-PINNs are a promising method to solve the inverse problem and to guide future experiments in bioengineering in the development of synthetic tissues and biosensors.^34^^,^^35^

## Results

### Pattern formation models

**Figure 1:**
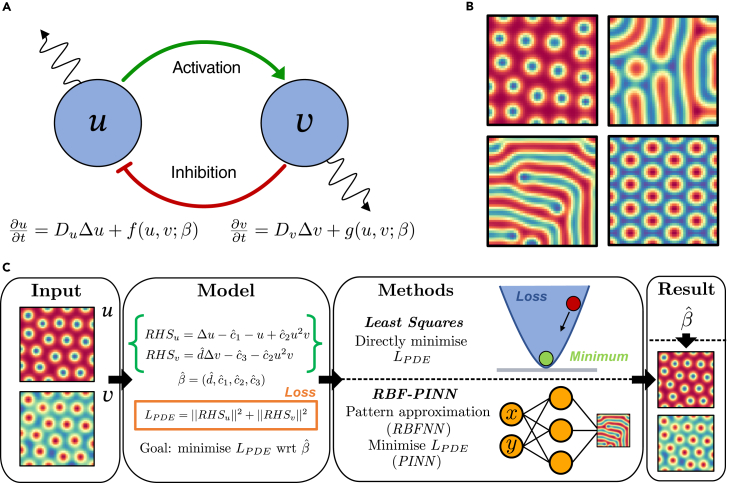
Overview of our methods for the Turing inverse problem (A) Network of the canonical Turing pattern with a short-range activator and a long-range inhibitor. (B) Turing patterns from different models: spots from the Schnakenberg model (top-left), labyrinths from FitzHugh-Nagumo (top-right), and labyrinths and spots from the Brusselator for two different parameter sets (bottom row). (C) Methodology of the paper: Starting from a Turing pattern, we build a PDE loss which is minimized with respect to the parameters β. This is done using two different methods, LS and RBF-PINNs, with best estimate $\hat{\beta }$. Once we have the best estimate, we assess the accuracy of the methods both by comparing the newly inferred parameters with the previous ones, and by comparing the patterns produced by both sets of parameters.

Both of our approaches, LS and RBF-PINNs, utilize discretized Turing patterns as inputs to infer the parameters of a candidate model. In this work, the candidate model refers to the true model that was numerically solved to produce the images using the finite-differences algorithm (see STAR methods section numerical simulations). We explored several models, all following the idea of activator-inhibitor dynamics shown in Figure 1A but before delving into specific models, we present the general two-component reaction-diffusion model for concentrations *u* and *v*, which depend on space and time, as follows:

(Equation 1) $$\begin{array}{l} u_{t}=D_{u}\Delta u+f\left(u,v\right)\text{,}\\ v_{t}=D_{v}\Delta v+g\left(u,v\right)\text{,} \end{array}$$

where $u_{t}$ and $v_{t}$ denote partial differentiation with respect to time and the functions f(u,v) and g(u,v) are non-linear reaction kinetics. Depending on the specific *f* and *g* functions, different models can be identified. Here, we will focus on three: First, the Schnakenberg model,^2^ which has a total of 6 parameters with 4 of them inside the functions *f* and *g* and the other two given by the diffusion coefficients:

(Equation 2) $$\begin{array}{l} f\left(u,v\right)=c_{1}-c_{2}u+c_{3}u^{2}v\text{,}\\ g\left(u,v\right)=c_{4}-c_{3}u^{2}v\text{.} \end{array}$$

Second, the FitzHugh-Nagumo model,^5^^,^^6^ which has 5 parameters:

(Equation 3) $$\begin{array}{l} f\left(u,v\right)=c_{1}\left(v-c_{2}u\right)\text{,}\\ g\left(u,v\right)=-u+c_{3}v-v^{3}\text{.} \end{array}$$

Third, the Brusselator model,^4^ with a total of 4 parameters:

(Equation 4) $$\begin{array}{l} f\left(u,v\right)=c_{1}-\left(c_{2}+1\right)u+u^{2}v\text{,}\\ g\left(u,v\right)=c_{2}u-u^{2}v\text{.} \end{array}$$

We numerically solve these PDE models on a square domain with no-flux boundary conditions for a closed system, resulting in patterns similar to Figure 1B. There are different kinds of patterns, and we will mostly focus on dots (Figure 1B, top left) and labyrinths (Figure 1B, top right). The type of pattern is mostly dependent on the model that we choose: FitzHugh-Nagumo produces labyrinths while Schnakenberg and Brusselator can produce both labyrinths and spots depending on the parameters. The parameters we used for the Brusselator and the FitzHugh-Nagumo models can be found in,^24^ while those for the Schnakenberg model are in.^36^ All parameters can also be found in Table S1. Although presented above as different, the Brusselator and the Schnakenberg model can be viewed as both specific cases of a more general 6-parameter model.^37^ Hence, most results relating to the former model can be found in Figures S1 and S2. In general, different initial conditions will produce different patterns of the same type, but fixing the initial conditions will produce the same pattern. (If two types of patterns are stable for the same parameters, the initial conditions may determine the type of pattern obtained.^23^) Hence, we can conceptually think of the parameters of the model and the initial conditions as the ‘variables’ that produce a given pattern. Since we are mostly interested in the parameters, we used the same initial conditions for all patterns. This eases the comparison between the patterns produced by different methods. Note that boundary conditions also play a role: for a discussion of initial conditions and boundary conditions, see STAR methods section initial and boundary conditions and Figure S4. As a result, there is a more direct relationship between the parameters and the patterns. The inverse problem consists of inverting this relationship by recovering the parameters that generate a given pattern, as outlined in Figure 1C. Nevertheless, we would like to emphasize that the fixing of the initial conditions is not a necessary assumption or condition for our methods; it is merely a tool to ease the comparison of the results. In fact, none of the methods need any knowledge of the initial conditions, since they are solely dependent on the final pattern.

### Non-dimensionalization for increased identifiability

To avoid trivial non-identifiability issues in the inverse problem, and with the added benefit of reducing the number of parameters in each model, we non-dimensionalized the three models (see STAR methods section non-dimensionalization for details). For the Schnakenberg model we obtain:

(Equation 5) $$u_{t}=\Delta u+c_{1}-u+c_{2}u^{2}v\;v_{t}=d\Delta v+c_{3}-c_{2}u^{2}v\text{,}$$

the FitzHugh-Nagumo model can be rewritten as:

(Equation 6) $$u_{t}=\Delta u+v-c_{1}u\;v_{t}=d\Delta v+c_{2}v-c_{3}u-v^{3}\text{,}$$

and the non-dimensional form of the Brusselator model is:

(Equation 7) $$u_{t}=d\Delta u+1-u+c_{1}u^{2}v\;v_{t}=\Delta v+c_{2}u-c_{1}u^{2}v\text{,}$$

where in all equations *d* is the ratio of the diffusion coefficients and the rest of the parameters are non-dimensional. Note that even though we have not changed the symbols of the parameters for simplicity of notation, they are different from the ones in Equations 2, 4, and 3. For the definition of the new variables and parameters of the three models, see STAR methods section non-dimensionalization and Table S1.

### LS interpretation of the problem

Our first approach to solving the inverse problem is based on fitting the parameters to the PDE equations, by fixing the concentrations *u* and *v* as given by the Turing pattern we aim to reproduce. If we assume a steady-state pattern of the PDEs, e.g., Equation 5, then $u_{t}=v_{t}=0$. We can now consider the RHS of Equation 5. Because we assume we have access to the discrete patterns u, v satisfying the PDE, we can fix them and treat the RHS as a function dependent only on the parameters, which will be zero for the combination of parameters that we want to find. Since our patterns are discrete in space (we can think of them as images with pixels), u and v are matrices, with their respective elements $u_{ij}$ and $v_{ij}$ for i,j=1,2,…,N and *N* the number of rows and columns in the pattern. This makes it possible to write and solve the problem using LS, as we can now formulate the problem as Xβ=Y, where *X* is the design matrix, β is the vector of parameters, and *Y* are all terms that are not parameter dependent. (For an example, see STAR methods section least squares.) This LS minimization is equivalent to minimizing the squared of the Frobenius norm (element-wise $L_{2}$ norm for matrices) for both equations, which we can write as:

(Equation 8) $$L\left(\beta \right)={\left\|\beta_{1}\Delta u+f\left(\beta \right)\right\|}_{F}^{2}={\left\|\Delta v+g\left(\beta \right)\right\|}_{F}^{2}\text{.}$$

The Laplacian is approximated using a second-order finite difference method, and the no-flux boundary conditions are taken to be the same as used to simulate the pattern. Since we assume the Turing pattern is at steady state, so that $u_{t}=0$, we refer to this method as a steady-state approximation.

**Figure 2:**
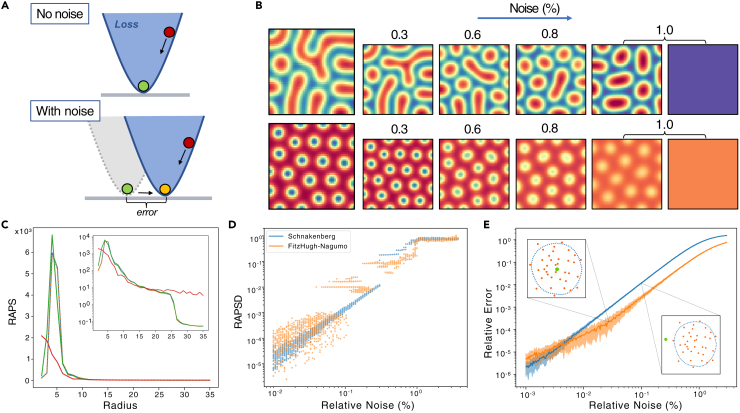
Least squares for parameter inference (A) In LS, noise changes the loss function that we minimize and the optimal value shifts, leading to some error in the inferred parameters. (B) Resulting patterns obtained from LS with different noise levels. After corrupting the original pattern with relative noise of different levels, parameters are obtained using LS and the model is solved with the inferred parameters to obtain the new patterns shown. The upper row corresponds to the FitzHugh-Nagumo model, and the lower row to the Schnakenberg model. The enlarged patterns on the left are the original ones. It can be observed that at 1% noise, the predicted patterns are noticeably different from the original ones across the models, and that we can obtain several patterns at the same level of noise. (C) Radially averaged power spectra (RAPS) obtained for different patterns recovered at different noise levels (shown in (B)) from the Schnakenberg model. Red corresponds to 1% noise, green to 0.6%, orange to 0.3% and blue is the original pattern. Inset shows the same plot but the *y* axis in log scale. (D) Scatterplot of RAPS differences for different relative noise levels with noticeable sudden increases due to discrete changes in the predicted Turing patterns. (E) Average relative error of inferred parameters for different relative noise levels in the Schnakenberg and FitzHugh-Nagumo models, with a sketch of the parameter space explaining the difference in spread. Green point represents the original set of parameter values and orange points are the different optimal sets resulting from the minimization. The biggest standard deviation in relative error occurs when the optimal sets are close to the original so the scale of the error changes drastically (can be very close or far), while the smaller standard deviation occurs when the optimal points are further away and the error is always on a similar scale.

To apply the LS method, we first select a model and a set of parameters, $\beta_{orig}$, that can produce a Turing pattern. Then, using the model and $\beta_{orig}$ we numerically solve the PDEs to produce a pattern similar to the ones shown in Figure 1B. Once we have the patterns, we write down the model in matrix form and find the best parameter combination using LS. This method produces very accurate results without noise in the pattern, with an average relative error in parameters of the order of $O\left(10^{-15}\right)$ or lower, which can be considered artifacts given its closeness to numerical precision. Nevertheless, when we corrupt the pattern with noise before applying LS, the ‘true’ minimum shifts from $\beta_{orig}$, producing an error which is no longer a numerical artifact (Figure 2A). The way we incorporate noise is by adding a matrix of normally distributed random variables with zero mean and varying standard deviation σ, relative to the concentration matrices u and. Since different u and v will have different ranges, the standard deviation of the noise must be relative to the range (maximum minus minimum value in the pattern) of each concentration matrix.^36^ To achieve this we employ what we denote ‘relative noise’. If we let $R_{p}\left(u\right)$ be the range of the concentration matrix u, so that

(Equation 9) $$R_{p}\left(u\right)=\max_{1\leq i,j\leq N}\left\{u_{ij}\right\}-\min_{1\leq i,j\leq N}\left\{u_{ij}\right\}\text{,}$$

We can define s% relative noise to correspond to standard deviation $\sigma =\frac{s}{100}R_{p}$. Once we add noise to the pattern, we can use these ‘noisy patterns’ as input for our methods, and by changing the level of noise we can systematically investigate the difference in performance and accuracy. This will yield a different parameter set for each noisy pattern, which can be used to solve the system and obtain a new pattern. The new patterns obtained and the respective level of noise from which their parameters were inferred are shown in Figure 2B. Before delving into our results, we remark that the figures only show the obtained results for the Schnakenberg and FitzHugh-Nagumo models. All the corresponding figures for the Brusselator model can be found in the supplemental information and will be referenced when relevant.

### LS performs poorly with noise

We consider two measures to assess the accuracy of our methods. First, the straightforward average relative error in parameters, which is fast to compute and easy to interpret, but has the drawback that the same relative error does not yield the same error in the produced patterns for different models. To remedy this, we used another measure based on the power spectrum of the pattern. We took the radially averaged power spectrum (RAPS) for each pattern, which is a curve as shown in Figure 2C, and we computed the mean squared error (MSE) between the curves of two different patterns. We will refer to this measure as RAPS difference or RAPSD for short. For a longer discussion of these measures see STAR methods section measuring similarity of patterns.

Using these two measures, we can analyze how LS performs for different levels of noise. As can be seen in Figures 2B and 2E for both the FitzHugh-Nagumo and Schnakenberg models and in Figures S1A and S2A for the Brusselator, at relative noise levels below around 0.5% we obtain very similar patterns, even when the corresponding relative error in parameters is around 0.1%. For larger levels of noise, both examples in Figure 2B begin to fail, resulting in parameters that do not produce patterns. Hence, we can observe that LS only produces reliable results up to 0.5–1% of relative noise. For a longer discussion of the behavior of LS with noise, see STAR methods section noise-limit of least squares.

If we consider the RAPS difference instead of the relative error in parameters, we observe a similar behavior. Instead of a continuous trend, we notice a more step-like behavior. This can be observed in Figures 2D and S2B, showing discrete jumps when comparing RAPS profiles with increasing noise levels. By comparing different noise levels in Figure 2B, we found that the last jump separates inferred parameters that produce a pattern from inferred parameters that do not. The other jumps are less clear and have to do with the patterns changing wavelength and scale. For example, for the Schnakenberg model, we can notice a modest linear trend, which shows that the range of the patterns is slowly changing. This is followed by a jump around 0.4%, which is the point where the spots have grown too much and the pattern changes to fewer dots (see the difference in spot number between 0.3% and 0.6% noise). For the FitzHugh Nagumo model, we notice more jumps and it is less clear what these represent. The general behavior is conserved for different patterns with the same parameters, but the position of jumps seems to be dependent on the initial conditions.

**Figure 3:**
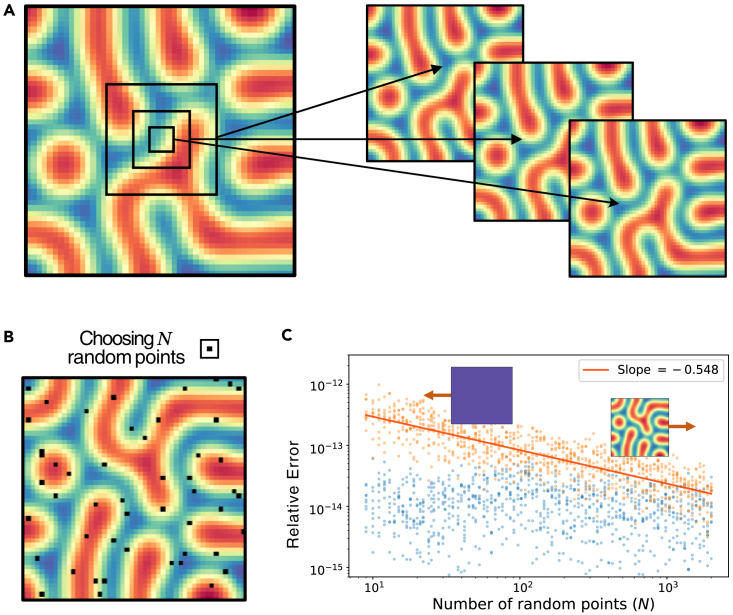
Effect of the number of pixels in the least squares method (A) Pattern from the FitzHugh-Nagumo model with three different cropped regions given by small squares of different sizes. It can be seen that a region of 3×3 pixels is sufficient to recover accurate enough parameters such that the predicted and original patterns (right and left respectively) are indistinguishable. (B) Schematic of choosing *N* randomly selected points (black) on the Turing pattern. (C) Effect of increasing the number of randomly selected pixels on the average relative error in the inferred parameters with (orange) and without (blue) added noise to the original pattern for the FitzHugh-Nagumo model. We used *N* in the range 5–2000 (50×50=2500 being the maximum possible) and sampled 10 different sets of pixels for each *N*, and we measured the relative error for each of the inferred parameters. There is almost no effect without noise, but with noise, there is a steady reduction in the relative error. Also shown is the slope of the line of best fit to the data (orange) and the dependence on the amount of sampled points: if we decrease the number of points enough, the parameter set does not produce a Turing pattern and a homogeneous steady state is reached instead (blue); if we increase the number, the approximation improves and we recover the Turing pattern.

These results show that the LS estimators are not robust to the levels of noise we aim for, but in the absence of noise, the method works well with little data. We tested this dependence on the amount of data by using a reduced region of the pattern, which can be seen in Figure 3A. We found that even if we crop the original pattern and use a 4×4 pixel region as input for the LS method, we still obtain the same patterns. A natural question is then to find the minimum number of pixels. Given the simplicity of the method, to answer this question we need only consider the formulation of LS. A full explanation is provided in STAR methods section least squares but we give a brief explanation here: given the design matrix *X*, which is determined by the model, we need $X^{T}X$ to be invertible. This condition will be satisfied when *X* has enough rows (a pixel corresponds to two rows) so that it is a full rank matrix. This means that the minimal number of pixels is model-dependent. For the Schnakenberg and Brusselator models, 2 pixels are enough, whereas 3 pixels were required for the FitzHugh-Nagumo model.

Next, we investigated how much the addition of new pixels changes the relative error in the parameters. To do this, we sampled *N* pixels from the pattern using a two-dimensional uniform distribution (Figure 3B), which we used to infer a new set of parameters. In Figure 3C we show a scatterplot of the mean relative errors for each N. We observed that the effect of the number of pixels on the relative error is different depending on whether the original pattern was corrupted with noise or not. Without noise (blue) we hardly see any improvement, but with noise (orange) there is a more drastic improvement in the parameters, which is expected as the noise has less effect the larger the sample. As the number of randomly selected pixels is increased, the relative error approaches that of no added noise with a power law, as can be seen in the log-log plot in the upper right corner of Figure 3C. The exponent of this power law is close to $-\frac{1}{2}$, which agrees with previous results^38^ on the LS convergence with *N*. For an intuitive mathematical explanation of this convergence, see STAR methods section power-law exponent. We remark that the fluctuations in relative error without noise (or for large *N* with noise) are numerical instabilities since they are also observed if we use all the data and merely change the order of the input. Similarly, we investigated this effect in the RAPSD for the Schnakenberg model in Figure S3. We saw that the observed behavior was dependent on the initial level of noise chosen: for around 0.03% noise, we observed a similar power law as with the relative error, with a slope again close to $-\frac{1}{2}$. For 0.25% noise, we observed the same jumps as can be seen in Figure 2D, with a convergence toward the same value shown in the figure for this level of noise. For more discussion of this case, see Figure S3.

We also attempted to apply LS to a design matrix defined by a model not used to generate the pattern. For example, we produce a pattern with the Brusselator model but we define the LS minimization using the Schnakenberg model. This is what we call ‘mixing’ the models, and the goal is to check if the models are flexible enough to produce the same pattern from one another. We found that this did not work even between models capable of producing the same type of pattern, and instead produced parameters that gave no Turing patterns at all (see the discussion section). In summary, the LS method works well without noise in the data, as it returns the exact parameters (with some numerical error), even when we have very little data (2–3 pixels depending on the model).

### RBF-PINNs are a noise-robust alternative

PINNs are a neural network architecture in which the network serves as a function approximator with two loss functions: the first compares the output of the network to the numerical simulation of the PDE (the pattern in our case) and makes sure the network outputs the correct values; the second tries to make the output of the network a solution of the PDE (the physical law) by both optimizing the network and optimizing the parameters of the PDE (the estimation).^32^ Using these two losses, the network approximates the solution as well as learns the optimal set of parameters for which the Turing pattern is a steady-state solution. This approach is computationally very efficient since it only adds a few parameters to the network which usually trains thousands. Hence, the extra computational cost is minimal, leading to excellent scaling with the number of parameters in the PDE. A peculiarity of this method is that if we have no noise in our input patterns, we can let the approximation overfit the data. This is because the approximation will become better which will improve the parameter optimization as well. Note, however, that this is no longer true when we consider noisy input.

**Figure 4:**
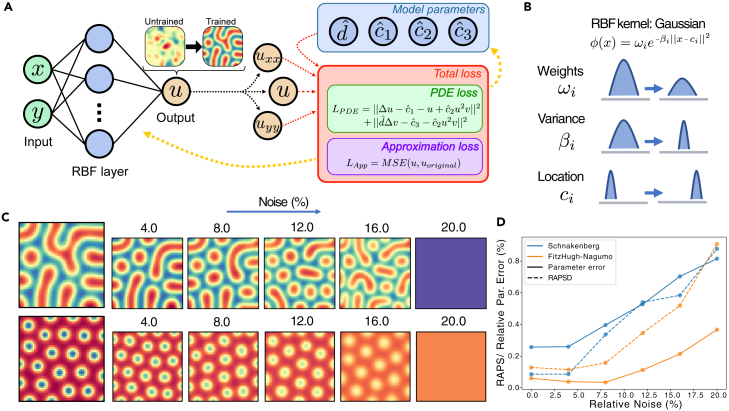
Physics-informed neural networks for parameter inference (A) Architecture of RBF-PINNs, where the input is space coordinates (x,y) and the output is the pattern. Input is shown in green, variables which are trained in blue and input to the losses in yellow. Red arrows denote usage in the loss and yellow arrows backpropagation. From the network, the partial derivatives can be efficiently computed using automatic differentiation and used in the PDE loss, where the PDE parameters are also network parameters. (B) Illustration of the three parameters of the Gaussian kernel and their interpretation. (C) Results from RBF-PINNs with different levels of added noise. After adding noise to the Turing pattern, the network is used to obtain a parameter set, which is subsequently used to predict the pattern. Patterns to the left are the original ones. (D) Relative error in the parameters and the RAPS difference for the respective parameters and patterns used in (C).

As function approximation, we used a radial basis function neural network (RBFNN), since it is especially suitable for data with regularities such as evenly spaced peaks and valleys like our Turing patterns. We used this network for its clear interpretability, but we also show in STAR methods section RBF-PINNs vs. traditional PINNs and Figure S6 that it performs better than a traditional PINN. This network only has one hidden layer (aside from input and output) defining the kernel. We can think of each kernel as adding a (unnormalized) distribution (Gaussian in our case) at a given location with a given variance and weight. These three are the only types of parameters of this network. Hence, the number of nodes is the number of kernels that we have, and during training these will change their location and variance, shrinking or growing to approximate the pattern. A representation of the three parameters and their effects are shown in Figure 4B.

As with our previous method, without added noise the network can approximate the pattern up to an arbitrary accuracy.^31^ This means that we can reach the same levels of accuracy as with the other method but with a caveat: we need to train the network for a long time to reach this level of accuracy and we would need a large number of nodes in the hidden layer. This is the reason behind the large error in Figure 4D without added noise since we use the same number of nodes for all noise levels. Note the performance can easily be improved without noise, but as our main focus is robustness to noise, we did not tune any parameters to any specific level of noise, except for the number of nodes and the variance of the kernels, and we used the same network structure. Once we apply noise, our results with this technique show an improved robustness when compared to LS, as can be seen in Figure 4C. For both FitzHugh-Nagumo (top) and Schnakenberg (bottom), we obtain very similar patterns up to a noise level of 12–16%. For the Brusselator model shown in Figure S1B, we can see similar results. Comparison with Figures 4D, S2C, and S2D shows that the relative error in parameters is higher in the Schnakenberg than in the FitzHugh-Nagumo model, but this error is much lower in the Brusselator model, indicating that this model is more robust to noise. Nevertheless, from Figure 4C we can see that there is not a direct relationship between relative error and similarity between the patterns (RAPSD), since for low noise levels (0–4%) the relative error in the Schnakenberg model is higher and the RAPSD is lower than the FitzHugh-Nagumo model. However, for higher levels of noise the RAPSD of the Schnakenberg model becomes higher. For convergence plots of some of the model parameters and losses of the network, see Figure S5 in supplemental information.

By looking at the RAPS profiles we find the same discrete jumps that we saw with the LS method. In particular, a well-pronounced final jump is observed, which we argued before indicates the transition from pattern to no pattern. By comparing Figures 2D and 4D, RBF-PINNs with 0–4% noise result in RAPS values corresponding to around 0.3–0.5% for LS, while RBF-PINNs with 8–12% noise correspond to around 0.6–0.9% for LS. This means that RBF-PINNs can infer parameters and predict patterns with around 10–20 times more noise than LS for the Schnakenberg and FitzHugh-Nagumo models. In the case of the Brusselator, this number is even higher, as can be seen by comparing Figures S2B and S2D, with RBF-PINNs being able to predict patterns with up to 40 times higher noise levels.

### Application of RBF-PINNs to experimental chemical patterns

Having demonstrated how inference by PINNs shows strong robustness to noise, the next step is to apply this method to experimental data. We chose chemical Turing patterns as an example because patterns are more stable and robust than biological patterns, and corresponding models are well established. Specifically, we consider the chlorine dioxide–iodine–malonic acid (CDIMA) system, used to study the impact of 2D growth on pattern formation.^9^ This reaction shows photosensitivity that was utilized to produce a time series of patterns in a radially growing domain using a mask. Since our method focuses on the steady-state pattern, we discarded all the time points except for the last one, focusing on the stable central region away from the boundary.

The experiments were modeled using the Lengyel-Epstein two-variable model,^39^ modified to incorporate the effects of illumination.^40^ Since we are only interested in the pattern, which is shown in the dark region, we will use the original model without illumination. We found that this model, although already non-dimensional, was problematic for parameter inference. This was because in one of the equations all terms have a parameter, which can lead to the new non-dimensionalized equations:

(Equation 10) $$u_{t}=d\Delta u+c_{1}-u-c_{2}\frac{4uv}{1+u^{2}},v_{t}=\Delta v+u-c_{2}\frac{4uv}{1+u^{2}}\text{,}$$

which we used to fit the data with our RBF-PINN. Before stating our results, there are a few complications worth mentioning. First and foremost, the scale of the pattern is unknown, since the original data is an image with pixel values ranging from 23 to 255. Secondly, the model depends on two concentrations (*u* and *v*), both exhibiting a pattern, while there is only one experimental pattern from emitted light. To solve both these problems, we define a free-scale variable *W*, which is a rescaled version of the original pattern in the [0,1] range. To obtain the model concentrations, we assume that there is a linear map from *W* to *u* and *v*, which we can write as:

(Equation 11) $$u=W\kappa_{u}+\gamma_{u},v=W\kappa_{v}+\gamma_{v}\text{,}$$

where $\kappa_{x}$ and $\gamma_{x}$ for x=u,v are scale and shift parameters respectively. The assumption of the existence of this linear map can be justified by the fact that patterns usually are either in phase (positive κ) or antiphase (negative κ). We further neglect that their widths might be slightly different.

**Figure 5:**
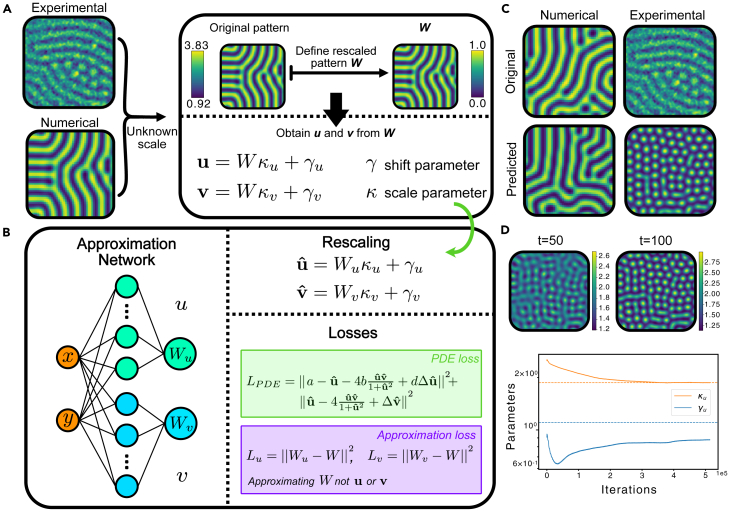
Application of RBF-PINNs to experimental chemical patterns (A) Explanation of scaling procedure, showing numerical and experimental patterns and how the scaling to the free-scale variables and the rescaling using the shift and scale parameters are performed. (B) Architecture of RBF-PINNs for the experimental case, with the division into the *u* and *v* approximation, the rescaling, and the different losses. (C) Results from RBF-PINNs to the numerical pattern (first column) and the experimental pattern (second column). The top images show the original patterns, while the bottom images show the predicted patterns using the inferred parameters and our numerical solver. (D) Time evolution of simulated experimental pattern showing how labyrinths are present at the initial time points and plot of convergence of scaling parameters.

As before, our network will consist of two independent subnetworks which will approximate *W* individually. From these two approximations, we will recover *u* and *v* using the scaling in Equation 11. Hence, we call these approximations $W_{u}$ and $W_{v}$, respectively. There are two reasons for using two approximations for the same variable. First, patterns might not be perfectly in phase or antiphase with each other. Second, the relationship between the scaled pattern and the true pattern might not be perfectly linear. Hence, by separating these into two variables we allow for flexibility in the method to make adjustments to each pattern individually. Another difference to the network used previously is that the scaling parameters are added as part of our PDE loss, and hence are optimized with the rest of the parameters, as portrayed in Figure 5B.

Our first attempt was using our inference method with numerical ‘data’. As before, the numerical simulation produced two patterns, but only one was used to increase the similarity with the experimental case. As a proof of concept, we started by fixing the scaling parameters to the best combination possible for both *u* and *v* given the simulated patterns, and obtaining the remaining parameters of the model. The predicted pattern is shown in the first column of Figure 5C (bottom), together with the original one (top). We can observe a good correspondence between them. Specifically, both show labyrinths on a similar scale. We obtained a RAPS of 0.2941 for the two numerical patterns, which is close to the value obtained for the FitzHugh-Nagumo model in Figure 4D below 16% of noise.

Encouraged by this numerical result, we attempted to apply our approach to the experimental pattern. We initialized all parameters close to the values from the numerical pattern and also used the same spatial dimensions. The results are shown in the second column of Figure 5C. We can observe that the predicted pattern (bottom) is not particularly close to the experimentally observed one (top). The obtained pattern also seems to show spots instead of the experimental labyrinths. Nevertheless, when inspecting the time evolution of this pattern in Figure 5D, we can observe that at the beginning the spots are connected and later on they move apart. By watching the experimental videos,^9^ we can actually observe this phenomenon too - at the beginning, we can only observe labyrinths but later on, spots begin to appear. This seems to point to the fact that the experimental pattern has not completely converged and that if left to evolve for longer, it would end up in a steady state with only spots. Of course, this begs the question how this affects the working of the model, since we assumed a steady-state pattern. Likely, the experimental pattern develops at a sufficiently low speed so that the time derivative is very close to zero. This could explain why the wave number of both experimental and numerical pattern do not fully match. Furthermore, in Figure 5D (bottom) we plot the convergence of the scaling parameters for *u* (solid lines), along with the values from the original numerical pattern (dotted lines). We can notice that they match quite well, with a slight mismatch of the shift parameter which is probably due to feedback in the training with other parameters. This feedback is caused because, when adding the scale parameters, the problem becomes underdetermined. Furthermore, we can infer that the experimental chemical pattern represents most likely the activator, $I^{-}$, which we later confirmed.^41^ This is because depending on whether the initial concentration belongs to *u* or *v* in the model, one non-dimensionalization will work better than the other; in our case only *u* worked. This result is further discussed in STAR methods section details on the modeling of chemical patterns.

In summary, our method was applied to experimental data with only minor manual intervention. Furthermore, we were able to obtain insight into the chemical pattern e.g., its closeness to a spotty steady state, due to the parameters we obtained from the method. Our method predicted the correct identification of the pattern with the activator chemical species.

## Discussion

We investigated two methods to solve the inverse problem in Turing patterns. The first was based on the least squares (LS) method, which we found to be very accurate with relative errors under $10^{-15}$, in noise-free patterns. We also proved that it works even with very small quantities of data. In fact, depending on the model Equations 2 and 3 pixels taken from the patterns were sufficient, assuming full knowledge of both concentrations and their Laplacians (see STAR methods section parameter identifiability). We also proposed another method based on neural networks, which we called RBF-PINNs since it is a mixture of PINNs and RBF neural networks. Without noise, the universal approximation theorem assures that we can approximate any function up to any given accuracy.^31^ The main advantage of RBF-PINNs is their suitability for noisy patterns, where our method was capable of recovering parameters close to the original ones, with up to 12–16% relative noise in the patterns. This allowed us to use this method for experimental chemical patterns, and obtain parameters that gave us new insight into the experimental patterns. For instance, our model predicts spots in the asymptotic limit and that observed patterns represent the activator species.

Each of these methods has their strengths and weaknesses. The main disadvantage of LS is its sensitivity to noise, common in biological data. This was remedied by our RBF-PINN method at the expense of a computational cost several orders of magnitude larger than LS, taking nearly an hour to train on an HP Z8 G4 Workstation. Interestingly, the inferred parameters without noise are not as accurate as LS, which can be observed by comparing the relative errors in Figures 2D and 4D. This performance can be further optimized by using more nodes in the network. Also, the robustness to noise can be improved. Both our approaches used a square grid of 50×50 pixels, but as we increase the number of pixels, the performance of both methods in the presence of noise becomes more robust. We can also run the optimization of the loss functions for longer to obtain better convergence, or alter the architecture to make it more specific to the pattern type. In this study we used the same network architecture for all patterns, only changing the variance of the starting RBF kernels since patterns have different wavelengths and scales, and the number of nodes.

When comparing with other methods for solving the inverse Turing problem, we can distinguish between approaches with and without noise. For the approaches without noise, our simple LS performs better than, e.g., Bayesian-based approaches,^24^ since LS does not require training and it is more generalizable, only relying on a single pattern and not a comprehensive library of patterns. However, LS may not work for experimental data while a Bayesian method might. Compared to the ones that did consider noise,^25^^,^^27^ the main difference is that we do not require knowledge of the initial conditions or transient data (dynamics). These are assumptions that would not hold for biological data, which usually is scarce with high levels of noise. Hence, our approach is, to the best of our knowledge, the first that tackles all of these issues, i.e., small data and noise, at the same time. There are some recent applications of neural networks to solve the inverse problem in reaction-diffusion equations, but with some limitations: PINNs applied to Turing patterns did not consider noise^42^ and a recurrent-neural-network-based architecture assumed extensive knowledge of the dynamics and was not tested on actual data.^43^

Although this work focused on finding the parameters of a predefined model given its output, identifying the model equations that can explain the data is another important question, also termed *model discovery*. Here, we had synthetic patterns in mind, where circuits are added to the cells, largely determining the model. However, we did test the model discovery methods of the *PySindy* package,^44^^,^^45^ including the ones using the weak formulation of the PDE model.^46^^,^^47^ We found that, although extremely useful in many applications, these methods are ultimately not applicable to our problem. This is because our patterns are at steady-state, which leads to the trivial solution with all terms having zero coefficients. Even if we use more than one time point, the time derivatives are too small and the methods still converge to the trivial solution. Furthermore, Turing models can be designed to produce certain types of patterns in specific regions of parameters space.^48^ Hence, the same pattern could potentially be produced with different models. This immense flexibility makes model recovery challenging.

While many models and parameters can produce the same type of pattern, e.g., spots or stripes, our work suggests that the details of the patterns are unique for the studied non-dimensionalized models with a given parameter set and knowledge of the spatial scale. Without noise, LS did not support mixing of models, in which a pattern from one model is attempted to be inversely solved by another. This is not a general statement and rather an observation based on the models considered. It would be interesting to consider other models and investigate if this mixing of models can be achieved. Note for LS to be well defined we require the design matrix to be of full rank, implying uniqueness of the solution for the parameters, that is, the identifiability of the parameters given a pattern. Hence, our LS results open up a new perspective on the Turing robustness problem: a pattern may act like a barcode to a specific parameter combination, given the key to the correct model.

In conclusion, our approaches open up new ways of connecting mathematical models to experimental patterns. In particular, RBF-PINNs worked for noise levels comparable to biological data and proved to be useful at elucidating properties of chemical patterns. Hence, this method could potentially be applied to infer parameters for biological candidate models, “proving” that a model is capable of reproducing observed patterns. This type of model confirmation could ultimately aid the rational design of synthetic tissues with patterns for downstream templating and added functionality.^49^^,^^50^ We hope our machine-learning approaches to solving inverse problems stimulate new research into unraveling pattern formation in biology, chemistry, and bioengineering.

### Limitations of the study

While PINNs address the noise limitation of LS, there are some limitations worth mentioning. First, we did not use domain growth for our work, which would be more biologically relevant. This could be implemented by considering more traditional PINNs that take time into account. However, this is outside the scope of the current study, focusing on steady-state patterns. Our approach to incorporating the scaling in the chemical patterns was found to be very sensitive to interference among parameters during training. A more sophisticated approach could potentially provide a better distinction in the parameters and yield more robust results. Looking at possible biological applications, a problem with Turing models is their simplicity, not matching the complexity of actual biological networks as, e.g., encountered in developmental biology. To inform biological experiments, future models need to be complex enough to allow meaningful connections to experimental observables, while being simple enough to avoid the ‘curse of dimensionality.’

## STAR★Methods

### Key resources table

**Table:** 

REAGENT or RESOURCE	SOURCE	IDENTIFIER
**Deposited data**		

Code used for analyses	This paper	https://github.com/Endres-group/IPTP-paper-code
Chemical patterns	Konow C et al., Turing patterns on radially growing domains: experiments and simulations. Phys Chem Chem Phys. 2019; 21:6718-2	https://doi.org/10.1039/C8CP07797E

**Software and algorithms**		

Python 3.9.16	Python	https://www.python.org
Tensorflow 2.11.0	Tensorflow	https://www.tensorflow.org/
Numpy 1.22.1	Numpy	https://numpy.org/
Maptlotlib 3.7.2	Matplotlib	https://matplotlib.org/
SciPy 1.9.3	SciPy	https://scipy.org/
opencv-python 4.9.0.80	OpenCV	https://opencv.org/
shapely 2.0.3	Shapely	https://shapely.readthedocs.io/en/stable/
Affinity Designer 2	Seriff	https://affinity.serif.com

### Resource availability

#### Lead contact

Further information and requests related to code should be directed to the lead contact, Robert Endres (r.endres@imperial.ac.uk).

#### Materials availability

This study is computational and did not generate new reagents.

#### Data and code availability

• This study did not produce a dataset nor is one necessary to reproduce these results, aside from the ones related to the experimental patterns. This dataset can be found in https://doi.org/10.1039/C8CP07797E. Initial conditions and code are provided and are sufficient for reproduction of any other results presented.
• The code used in the analysis can be found in the GitHub repository: https://github.com/Endres-group/IPTP-paper-code.
• Any additional information required to reanalyse the data reported in this work is available from the lead contact.

### Method details

#### Technical specifications

All our algorithms and methods were written in Python 3.9.16, and the packages used depend on the method. For the LS method, the main packages we used was NumPy 1.22.1 and SciPy version 1.9.3. For the RBF-PINNs, we also used Tensorflow 2.11.0 to build and run the networks. All code was ran in an HP Z8 G4 Workstation with a Intel Xeon(R) Gold 6128 CPU @ 3.40 GHz × 24 processor and a Quadro RTX 6000/PCIe/SSE2 GPU.

#### Non-dimensionalization

Depending on the model, the inverse problem described above may be unsolvable because it may have many solutions. For example, take the Schnakenberg model from Equation 2 and assume *u* and *v* are steady-state patterns in time satisfying the PDE (Equation 1) for a given set of parameters. As a result, both the left-hand side (LHS) and right-hand side (RHS) of the PDE are zero. If we multiply all parameters by a constant *k* and substitute them back in the equation, the resulting set of parameters is also a solution to the inverse problem; since we can take *k* as a common factor, we again obtain RHS=0. Furthermore, if we let k=0 we arrive at a trivial solution where all parameters are zero. To avoid this, one remedy is to fix some parameters so that there is only a single solution. However, we found that non-dimensionalizing the equations is preferable since this does not require us to make any assumptions about the parameters. As an added benefit, this reduces the number of parameters of the model.

In this part, we outline the change of variables used to derive the non-dimensionalized formulas of the model equations we used. For each of the models, we firstly write the original model equations and the change of variables we derived for u,v,t and *x*. Subsequently, we give the change in the parameters that this variable change yields, denoting with a hat symbol the ‘new’, dimensionless variables and parameters. Finally we write the non-dimensional equations.

Using the same notation as before, we can write the Schnakenberg model as:

(Equation 12) $$\begin{array}{l} u_{t}=D_{u}\Delta u+c_{1}-c_{2}u+c_{3}u^{2}v\\ v_{t}=D_{v}\Delta v+c_{4}-c_{3}u^{2}v \end{array}$$

The original parameter set that we used is $(D_{u}\text{,}$ $D_{v}\text{,}$ $c_{1}\text{,}$ $c_{2}\text{,}$ $c_{3}\text{,}$ $c_{4})=(1\text{,}$ 40, 0.1, 1, 1, 0.9). To non-dimensionalize these equations, we can define the new variables $(\hat{u}\text{,}$ $\hat{v}\text{,}$ $\hat{t}\text{,}$ $\hat{x})=(\frac{c_{4}}{c_{2}}u$, $\frac{c_{4}}{c_{2}}v$, $c_{2}t\text{,}$ $\sqrt{\frac{c_{2}}{D_{u}}})$. This gives the new parameter set: $(\hat{d}\text{,}$ ${\hat{c}}_{1}\text{,}$ ${\hat{c}}_{2}\text{,}$ ${\hat{c}}_{3})=\frac{D_{v}}{D_{u}}$, $\frac{c_{2}c_{3}}{c_{1}^{2}}$, $\frac{c_{4}c_{1}}{c_{3}^{2}}$, $\frac{c_{2}c_{1}}{c_{1}c_{2}}=1)$. Substituting this into Equation 12 gives us:

$${\hat{u}}_{t}=\Delta \hat{u}+{\hat{c}}_{1}-\hat{u}+{\hat{c}}_{2}{\hat{u}}^{2}\hat{v}$$

$${\hat{v}}_{t}=\hat{d}\Delta \hat{v}+{\hat{c}}_{3}-{\hat{c}}_{2}{\hat{u}}^{2}\hat{v}$$

The equations for the FitzHugh-Nagumo model are:

(Equation 13) $$\begin{array}{l} u_{t}=D_{u}\Delta u+c_{1}\left(v-c_{2}u\right)\\ v_{t}=D_{v}\Delta v-u+c_{3}v-v^{3} \end{array}$$

The parameter set used was $(D_{u}\text{,}$ $D_{v}\text{,}$ $c_{1}\text{,}$ $c_{2}\text{,}$ $c_{3})=(0.05\text{,}$ 0.00028, 10, 1, 1). The non-dimensionalization that we found is given by the new variables $(\hat{u}\text{,}$ $\hat{v}\text{,}$ $\hat{t}\text{,}$ $\hat{x})=(\sqrt{c_{1}}u$, $\sqrt{c_{1}}v$, $c_{1}t\text{,}$ $\sqrt{\frac{c_{1}}{D_{u}}})$. This gives the new parameter set: $(\hat{d}\text{,}$ ${\hat{c}}_{1}\text{,}$ ${\hat{c}}_{2}\text{,}$ ${\hat{c}}_{3})=(\frac{D_{v}}{D_{u}}\text{,}$ $c_{2}\text{,}$ $\frac{c_{3}}{c_{1}}\text{,}$ $\frac{1}{c_{1}})$, which after substituting into Equation 13 gives:

$${\hat{u}}_{t}=\Delta \hat{u}+\hat{v}-{\hat{c}}_{1}\hat{u}\text{,}$$

$${\hat{v}}_{t}=\hat{d}\Delta \hat{v}+{\hat{c}}_{2}v-{\hat{c}}_{3}\hat{u}-{\hat{v}}^{3}$$

#### Numerical simulations

All numerical simulations of the models were performed on a 50×50 discrete grid by applying a center difference approximation to the second-order derivatives in the Laplacian, hence transforming the PDE Equation 1 into an ODE model:

(Equation 14) $$\begin{array}{l} \frac{du_{i,j}}{dt}=D_{u}\frac{u_{i,j-1}+u_{i,j+1}+u_{i-1,j}+u_{i+1,j}-4u_{i,j}}{{\left(\Delta x\right)}^{2}}+f\left(u_{i,j},v_{i,j}\right)\text{,}\\ \frac{dv_{i,j}}{dt}=D_{v}\frac{v_{i,j-1}+v_{i,j+1}+v_{i-1,j}+v_{i+1,j}-4v_{i,j}}{{\left(\Delta x\right)}^{2}}+g\left(u_{i,j},v_{i,j}\right)\text{.} \end{array}$$

Here, we assumed that the step in the *x* direction is the same as the step in the *y* direction, i.e., Δx=Δy. The initial condition used corresponds to the homogeneous steady state of the PDE without the Laplacian plus noise. We used *iid* Gaussian noise added pixel-wise. We saved this noise array for easier comparison with other models, which can be found in the repository for the code. Using this initial condition together with Equation 14, we use solve_ivp with the BDF solver for stiff problems. This allows us to forward integrate these equations for a sufficiently large amount of time, ensuring that the system has converged to a steady-state Turing pattern. We also define a criterion for early convergence, triggered when the $L_{2}$-norm of the derivative matrix is sufficiently small (less than $10^{-12}$).

#### Initial and boundary conditions

Throughout the text we mention that we keep the same initial conditions to have an easier visual comparison and to be able to quantitatively measure how well the resulting parameters from our methods reproduce the patterns produced with the original parameters. Likewise, we also use a single type of boundary condition. Here, we emphasize and support the fact that the performance of our methods does not depend significantly on the choice of initial conditions or boundary conditions. In Figure S4A, we show the relative error of LS parameter estimation as a function of the relative noise added, for five different initial conditions, each generating a different pattern. It can be seen that the trend is the same for each initial condition, since the mean values, although fluctuating (due to the noise added) are all overlapping. Similarly, in Figure S4B we can see the same plot but using patterns generated with Neumann (blue) and periodic (orange) boundary conditions. As before, we can see that for small noise, the two patterns show the same trend, but as we increase noise, we can see that the method produces a lower relative error with the pattern generated using periodic boundary conditions. This could be due to extra symmetries that the periodic boundary conditions impose.

From both of these plots, we can see that the initial and boundary conditions do not change drastically the accuracy of LS at recovering the parameters. We want to emphasize here that none of our methods make use of initial conditions, which is why it makes sense that the performance does not change, since even if we change the initial conditions we can expect the final patterns to be very similar to each other. As discussed above, this could be different if the parameters were in a region where initial conditions determine the type of parameter,^23^ but in general, this is not the case. For the boundary conditions, both of our methods discard a region of the boundary (usually 3–5 pixels wide) and in this way, we do not need knowledge or make any assumptions about the type of boundary condition that produced the pattern. This is because, in general, this might not be known for a given pattern, and our primary interests are the parameters of the model. This does not mean that changing the boundary conditions does not change the results of our methods, as we saw in Figure S4B, since different boundary conditions will constrain the patterns produced in different ways, which may make the parameter inference harder or easier.

#### Measuring similarity of patterns

We considered several measures to assess the accuracy of our method. First, we looked at the average relative error in the parameters, which gives us a measure of how close the newly obtained parameters are to the original ones. A drawback of this measure is that, depending on the model, very distinct parameters can give us the same pattern, or conversely similar parameter values can yield very different patterns. Hence, a better measure for parameter accuracy would be one that quantifies how similar the pattern produced with an inferred parameter vector is to the original pattern. We found that measures like the MSE are not very useful, since we do not expect the patterns to be exactly the same. This is because our main objective is to be able to recover the parameters of the model, but the final pattern is not determined solely by the parameters, but also by the initial conditions; the parameters and model determine the type of Turing pattern and its wavelength, but the initial conditions determine the position and shape. There are some cases^23^ where two patterns might be stable simultaneously, so initial conditions might change the patterns. Hence, we do not emphasize obtaining the exact positions and shapes. In order to focus on the type of pattern and wavelength, we instead compare the patterns in the frequency domain. Specifically, we use the Fourier power spectrum and take the radial average to obtain a one-dimensional profile which should have a main dominant frequency for each of our patterns. This is called radially averaged power spectrum (RAPS). Example RAPS curves for different profiles are shown in Figure 2C. Then, we can compute this RAPS for each pattern and use the MSE between these two profiles as a similarity measure. We refer to this measure as RAPS difference or RAPSD for short.

Another key difference between these two measures is that the relative error does not depend on solving the forward problem, while the RAPSD does. This means that in a situation where two patterns are stable, the RAPSD might not produce consistent results since initial conditions might change the pattern produced. Hence, we would have to rely on the relative error in parameters to assess the accuracy of the methods.

#### Least squares

As described in the section on LS, to write down the solution we first need to translate our problem into matrix form. We will do this here with the Schnakenberg model as an example. Using the same notation as before, we let $u_{ij}$ and $v_{ij}$ be our discretized Turing patterns in matrix form, for i,j=1,2…,N, with *N* being the number of columns and rows (as we have a square grid). Writing Equation 5 using this notation and at steady state, we obtain:

(Equation 15) $$u_{ij}-\Delta u_{ij}=c_{1}+c_{2}u_{ij}^{2}v_{ij},0=d\Delta v_{ij}+c_{3}-c_{2}u_{ij}^{2}v_{i,j}\text{,}$$

where we collected the terms not multiplied by a parameter to the left. Note that this is simply a linear system of $2\times N^{2}$ equations, which we can write in matrix form as:

$$\left(\begin{array}{c} u_{11}-\Delta u_{11}\\ u_{12}-\Delta u_{12}\\ \vdots \\ u_{NN}-\Delta u_{NN}\\ 0\\ 0\\ \vdots \\ 0 \end{array}\right)=\left[\begin{array}{cccc} 1 & u_{11}^{2}v_{11} & 0 & 0\\ 1 & u_{12}^{2}v_{12} & 0 & 0\\ \vdots & \vdots & \vdots & \vdots \\ 1 & u_{NN}^{2}v_{NN} & 0 & 0\\ 0 & -u_{11}^{2}v_{11} & 1 & \Delta v_{11}\\ 0 & -u_{12}^{2}v_{12} & 1 & \Delta v_{12}\\ \vdots & \vdots & \vdots & \vdots \\ 0 & -u_{NN}^{2}v_{NN} & 1 & \Delta v_{NN} \end{array}\right]\left(\begin{array}{c} c_{1}\\ c_{2}\\ c_{3}\\ d \end{array}\right)$$

We write this equation as y=Xβ, and call *y* the vector of independent variable and *X* the matrix of dependent variables. If we look at this problem from a regression perspective, it is customary to call *y* the vector of outputs and *X* the design matrix. With enough elements, this is an overdetermined system of linear equations. This means it will not have an exact solution in general, but we can obtain the best approximation using the LS formulation.^29^ By defining the error vector and finding its minimum, the solution for the optimal parameters can be written as:

$$\beta ={\left(X^{T}X\right)}^{-1}X^{T}y\text{.}$$

#### Parameter identifiability

Note that for this problem to be well defined we need $X^{T}X$ to be invertible, which is equivalent to *X* having full rank. Not only is this a necessary condition for LS, but also for the uniqueness of the solution for the parameters, that is, the identifiability of the parameters given a pattern. Based on this necessary condition, we can obtain the minimum amount of pixels needed for the solution to be well defined. For the Schnakenberg model, we find that two pixels are enough since that provides a 4×4 matrix in the form:

$$M=\left[\begin{array}{cccc} 1 & a & 0 & 0\\ 1 & b & 0 & 0\\ 0 & -a & 1 & c\\ 0 & -b & 1 & d \end{array}\right]\text{,}$$

which for most a,b,c,d is full rank. There are cases (e.g., c=d or a=b) for which *M* is not full rank. This is partially due to symmetries in the pattern but with enough randomly selected pixels in the pattern, such cases are unlikely. Taken together, this condition leads to the requirement of two pixels for the Brusselator model (due to two independent 2×2 submatrices) and three for the FitzHugh-Nagumo model (due to a 3×3 submatrix). Note that here we assume that these pixels have correct values, including for the Laplacian. This means that there is also local information on the pixels’ neighbors. Hence, effectively we use more information than the least amount of pixels.

#### Noise-limit of least squares

Here, we describe further details on how the noise affects the LS estimator. In Figure 2E we can see that the standard error in parameters (the shaded region encompassing the solid lines) behaves very differently for the two models considered, and its width seems to reduce at different values of relative error, so the scale of the error does not cause it. We found that the point where this error is reduced is when the LS results are not centered around the original set of parameters anymore. To visualize this, it is helpful to note that the LS estimator is dependent on noise. Hence, different realizations of the corrupted pattern at the same level of noise will produce different LS estimators, which will produce different patterns, as can be seen with 1% noise in Figure 2B. We can either obtain a pattern or a constant solution, showing how the obtained parameters are at the boundary of the Turing region. These different estimators can be thought of as a set of points in parameter space. Subsequently, the reduction in standard error occurs as the set of points moves further away from the original set of parameters, as described in the sketch in Figure 2E). Note that since we are using LS, instead of computing the relative error in $\hat{\beta }$, it could be argued that we can use the statistical properties of the LS estimator and obtain an expression for its variance. Indeed, the relative error we are computing is proportional to the standard deviation of. Hence, if we had an expression for the variance, then the errors would be easily computed. However, this is not an easy task since the usual LS assumptions do not hold in our case (noise is not additive and does not have a constant variance, and the design matrix is noisy). Applying the statistical properties of the LS estimator results in errors that do not match our data, so we decided to numerically estimate the relative error.

#### Power-law exponent

Our measure of ‘relative error’ is the absolute error of the estimated parameters, which is the standard deviation of the distribution of the LS estimator divided by the parameter. We then computed the mean of this quantity. To explain the observed slope of the power law between this mean relative error and the number of randomly selected points in the pattern, we can consider the variance of the LS estimator,^29^ $var\left(\hat{\beta }\right)={\left(X^{T}X\right)}^{-1}\sigma^{2}$, where $\hat{\beta }$ is our parameter estimator, *X* our design matrix and $\sigma^{2}$ the variance of the noise. Although we indicated that this value did not match our results, we found that the rate of change of the variance as noise increased was approximately the same. While the main assumptions of additive and normally distributed noise are not fulfilled (e.g., our design matrix is noisy), this does not seem to affect this rate of change very much. Hence, we attempt to use the variance expression for this case. By assuming a constant variance, to obtain an expression for our mean relative error we would need to look at the square root of the diagonal elements of ${\left(X^{T}X\right)}^{-1}\sigma^{2}$, which are the standard deviation of each parameter. Since we can regard the mean relative error as the mean of the standard deviations of the parameters divided by the parameters themselves, we only need to divide by the parameters and then compute the mean to have an expression for the mean relative error:

(Equation 16) $$\text{Mean Relative Error}=\frac{1}{p}\sum_{i=1}^{p}\frac{{\left[{\left(X^{T}X\right)}^{-1}\sigma^{2}\right]}_{ii}^{1/2}}{\beta_{i}}$$

The only elements in this expression that depend on the number of pixels chosen are the diagonal elements of ${\left(X^{T}X\right)}^{-1}$. For a fixed *N*, $X^{T}X$ will be a p×p matrix, where *p* is the number of parameters. Since we are sampling the same pattern, we can approximate ${\left(X^{T}X\right)}_{ij}=\sum_{m=1}^{N}X_{im}X_{mj}=O\left(N\right)$, since each element of the matrix is a sum of *N* products and each product can be considered O(1) in *N* since they are not dependent on *N*. By substituting this in Equation 16 we obtain the $N^{-1/2}$ observed in the power law.

#### Network description

For the neural network, as described in the results section, we combined PINNs (i.e., using the PDE equations to improve the approximation and having the parameters as trainable variables in the network) with RBF neural networks to obtain what we called RBF-PINNs. The architecture is very simple since the network is made of only three layers. The input of the network is a vector representing a spatial location, in our case (x,y), since we only have two dimensions, and the output is u(x,y). We have one network for each *u* and *v*, to allow for more flexibility in the model, but we train them together. Between the input and the output layer, we have a single hidden layer, with an activation layer representing the RBF kernels, and the input being fed directly to this activation layer by having all weights set to 1. This means that the amount of trainable parameters is of the order of 3M with *M* the total number of nodes in the network. These RBF kernels can be written as:

(Equation 17) $$\varphi_{i}\left(x\right)=w_{i}e^{-\beta_{i}{\left\|x-c_{i}\right\|}^{2}}\;\text{for}\;i=1,2,\ldots \;M$$

As explained previously and portrayed in Figure 4, $\beta_{i}$ works as the variance, $c_{i}$ is the location of the $i^{th}$ kernel, and $w_{i}$ its weight or importance. Each node in the network represents a different kernel. Hence, the only way to change the architecture of the network to improve the accuracy of the approximation is to increase the number of kernels by increasing the number of nodes. The kernel parameters were initialized using a 2D uniform random variable in the spatial range of the pattern for c and the network weights were initialized using the customary Glorot initialization.^51^ The variance was initialized depending on the pattern since the variance of the Gaussian kernels has to be on a similar scale as the pattern wavelength.

Our network has two main losses: One is an approximation loss from comparing the output of the network to the pattern. If we consider our network as a function, we can write it as $\Phi^{u}\left(X\right)$ where X is the space dimensions with pattern values u. From now on we just write this as $\Phi^{u}$ and similarly for v. Subsequently, we can write the approximation loss for u as:

(Equation 18) $$L_{app,u}=\frac{1}{N^{2}}\sum_{i,j}{\left\|\Phi_{i,j}^{u}-u_{i,j}\right\|}^{2}\text{.}$$

Note that this is equivalent to computing the mean squared error (MSE) between the data and the approximation. We mentioned the RAPS loss before, and it could be used here for the network training as well, as it could help to cancel noise. However, since we care about the pattern being accurate, the MSE is preferred.

The second loss is the PDE loss, which enforces that the output has a small PDE residual and at the same time improves the parameters so that the data provides a better fit to the equation:

(Equation 19) $$L_{PDE}=\frac{1}{N^{2}}\sum_{i,j}{\left\|\beta_{1}\Delta \Phi_{i,j}^{u}+f\left(\Phi_{i,j}^{u},\Phi_{i,j}^{v},\beta \right)\right\|}^{2}+{\left\|\Delta \Phi_{i,j}^{v}+g\left(\Phi_{i,j}^{u},\Phi_{i,j}^{v},\beta \right)\right\|}^{2}\text{.}$$

We also have a loss for the diffusion term, to make sure that the second derivatives of the approximation are accurate. The final structure used for the results in the paper has 60–120 nodes depending on the model. For training, we use batch training with batches of 128 elements for 200,000 iterations and Adam optimizer, taking a total of less than an hour to run. The first 10,000–20,000 iterations are used to approximate the pattern, since our loss function only takes into account $L_{app}$. After this first portion of iterations, the pattern can be assumed to be well approximated. Hence, we add $L_{PDE}$ to the loss so that we subsequently train the parameters to find values that match the trained pattern. This needs to be done carefully, since $L_{app}$ is presumably very low as the network is already minimized, making $L_{PDE}$ much larger in comparison. Hence, we need to add a weight to one of the losses to put them on the same scale. We also emphasize here that we do not use the whole pattern for both losses. When training the approximation, we use all of the pixels in the pattern, but when we add the $L_{PDE}$, we only use interior points so that inaccuracies in the diffusion do not present a problem. To do this, we drop the 3–5 pixels nearest to the boundary. Hence, instead of having a 50×50 square pattern, we end up with one of size 44×44.

The structure of our network is relatively simple and the RBF network guarantees a smooth output. However, to avoid overfitting we can use a small number of nodes or kernels. This is because overfitting is only a problem for the approximation part of our network (that is where our data is used) and when we use a noisy pattern. Furthermore, our patterns have a larger wavelength than the noise. By selecting a variance or amplitude parameter to be of the same order as our pattern, we keep the network from incorporating most of the noise. On top of that, we redefine the weights of the network every 2,000 iterations so that all of them are on a similar scale. This is useful since some weights may converge faster than others, and as a result, lead to a very small loss if the original weights are used.

#### RBF-PINNs vs. traditional PINNs

We compared the performance of our RBF-PINNs with a vanilla PINN implementation, consisting of an MLP with 5 hidden layers of 80 nodes each, and with the *tanh* activation function. From the plots in Figure S6, we can see that both for relative error in parameters and RAPSD value, our implementation of RBF-PINNs works better than the traditional PINNs, while being more interpretable. Furthermore, the number of parameters used by the MLP is around 80×(80∗4+2)=25.760, while the number of parameters used by the RBF-PINNs is in the order of 80×4=360, which means RBF-PINNs use two orders of magnitude less parameters than the MLP.

#### Details on the modeling of chemical patterns

In this section we provide extra details for the inference using data from chemical patterns. Firstly, we will describe the models. As explained above, our starting model is the Lengyel-Epstein (LE) two-variable model.^39^ This model can be written as:

(Equation 20) $$u_{t}=D_{u}\Delta u+c_{1}-c_{2}u-c_{3}\frac{4uv}{s+u^{2}}\;v_{t}=D_{v}\Delta v+c_{2}u-c_{3}\frac{4uv}{s+u^{2}}\text{,}$$

The nondimensional form used in the paper from where experimental images were taken^9^ is based on earlier work,^39^ but as already mentioned we used a different non-dimensionalization, which is given by the change of variables $\hat{u}=\frac{u}{\sqrt{s}}$, $\hat{v}=\frac{v}{\sqrt{s}}$, $\hat{t}=c_{2}t$ and $\hat{x}=x\sqrt{\frac{c_{2}}{D_{v}}}$. As a result, the new parameters are given by ${\hat{c}}_{1}=\frac{c_{1}}{c_{2}\sqrt{s}}$, ${\hat{c}}_{2}=\frac{c_{3}}{c_{2}\sqrt{s}}$, $\hat{s}=s$ and $d=\frac{D_{u}}{D_{v}}$. With this, we can write the new equation as:

(Equation 21) $${\hat{u}}_{t}=d\Delta \hat{u}+{\hat{c}}_{1}-\hat{u}-{\hat{c}}_{2}\frac{4\hat{u}\hat{v}}{1+{\hat{u}}^{2}}\;{\hat{v}}_{t}=\Delta \hat{v}+\hat{u}-{\hat{c}}_{2}\frac{4\hat{u}\hat{v}}{1+{\hat{u}}^{2}}\text{,}$$

Note that if instead of letting $\hat{x}=x\sqrt{\frac{c_{2}}{D_{v}}}$ we let $\hat{x}=x\sqrt{\frac{c_{2}}{D_{u}}}$, we change the diffusion ratio to $d=\frac{D_{v}}{D_{u}}$ and now it appears in the second equation:

(Equation 22) $${\hat{u}}_{t}=\Delta \hat{u}+{\hat{c}}_{1}-\hat{u}-{\hat{c}}_{2}\frac{4\hat{u}\hat{v}}{1+{\hat{u}}^{2}}\;{\hat{v}}_{t}=d\Delta \hat{v}+\hat{u}-{\hat{c}}_{2}\frac{4\hat{u}\hat{v}}{1+{\hat{u}}^{2}}\text{,}$$

Although these two versions of the LE are very similar, since the only differences are the spatial scale and the value and location of *d*, we found that using one or the other could yield very different results. The reason for this is better explained by looking at the numerical procedure to test our method. We numerically solve the Equation 21 or 22 to produce patterns *u* and *v*. We then discarded one of these and scaled the remaining one so that we could obtain both an approximation for both *u* and *v* only from this. As mentioned, this relied on the assumption that the pattern solutions are perfectly in phase or out of phase so that a linear mapping can be defined. However, for numerical simulations and experiments the patterns may not be in phase (or out of phase) perfectly, because of discretization of our spatial domain. This means that one of the approximations will be more accurate than the other, and this was found to be a problem when inferring the diffusion ratio. Since this parameter is multiplied by the Laplacian of one of the concentrations, its inference is very sensitive to inaccuracies in the Laplacian so we are likely to obtain a wrong parameter set. Our solution is the following: if the pattern discarded is *u*, then we use the model in which the diffusion ratio is multiplying the Laplacian of *u* (i.e., Equation 21) and vice versa. Furthermore, if the identity of the original pattern is unknown, as is the case for the chemical patterns, we can try both models and see which one gives a more reasonable parameter set. Applying both models to the chemical patterns, we found that the only model which produced realistic (non-negative) parameters is Equation 21. This means that the concentrations in the pattern seem to be more related with the *u* concentration in the model, which is the activator species $I^{-}$ (iodide).^41^

Another problem that we found during training of the network is that by adding the scaling parameters κ,γ, the roles of these parameters are possibly mixed. As an example, we can look at the terms ${\hat{c}}_{1}-\hat{u}$ in the first equation of the model 21. By substituting $\hat{u}=W\kappa_{u}+\gamma_{u}$, we get ${\hat{c}}_{1}-W\kappa_{u}+\gamma_{u}$, and $\gamma_{u}$ and ${\hat{c}}_{1}$ can be considered to have the same role - they are both basal rates of production. Of course, $\gamma_{u}$ is also present in other terms which means this effect will be lessened, but other combinations of parameters also show this feedback. Hence, it is difficult to train the network from completely wrong parameters. For this reason, we start with initial values given by a parameter set from previously published numerical simulations.^9^ Although we start with these values, we do end up with a different parameter set, and the scaling parameters obtained from the network agree with the scale of the patterns obtained from the inferred parameters.

### Quantification and statistical analysis

A characteristic of our methods is that they require only a single image of the pattern. Next, we summarize some statistical terminology used in the text. We define the relative noise in Equation 9 as a percentage relative to the spread of the pattern, that is, the maximum value minus the minimum. The error used to quantify how different new parameters are from the old ones is the mean relative error, that is, for each parameter we compute the absolute value of the difference between the old and new parameter values, and we take the mean of the differences for all parameters. The noise added to the patterns is a matrix of *iid* normally distributed random variables.

## Supplemental information

Document S1. Figures S1‒S6 and Table S1
