# Supplementary material for: Unraveling biochemical spatial patterns: Machine learning approaches to the inverse problem of stationary Turing patterns
Source: iScience. 2024 Apr 29;27(6):109822. doi: 10.1016/j.isci.2024.109822 (PMC11140185; doi:10.1016/j.isci.2024.109822)
Supplement: Document S1. Figures S1‒S6 and Table S1 [file mmc1.pdf]

iScience, Volume *27*

Supplemental information

Unraveling biochemical spatial patterns: Machine learning approaches to the inverse problem of stationary Turing patterns

Antonio Matas-Gil and Robert G. Endres

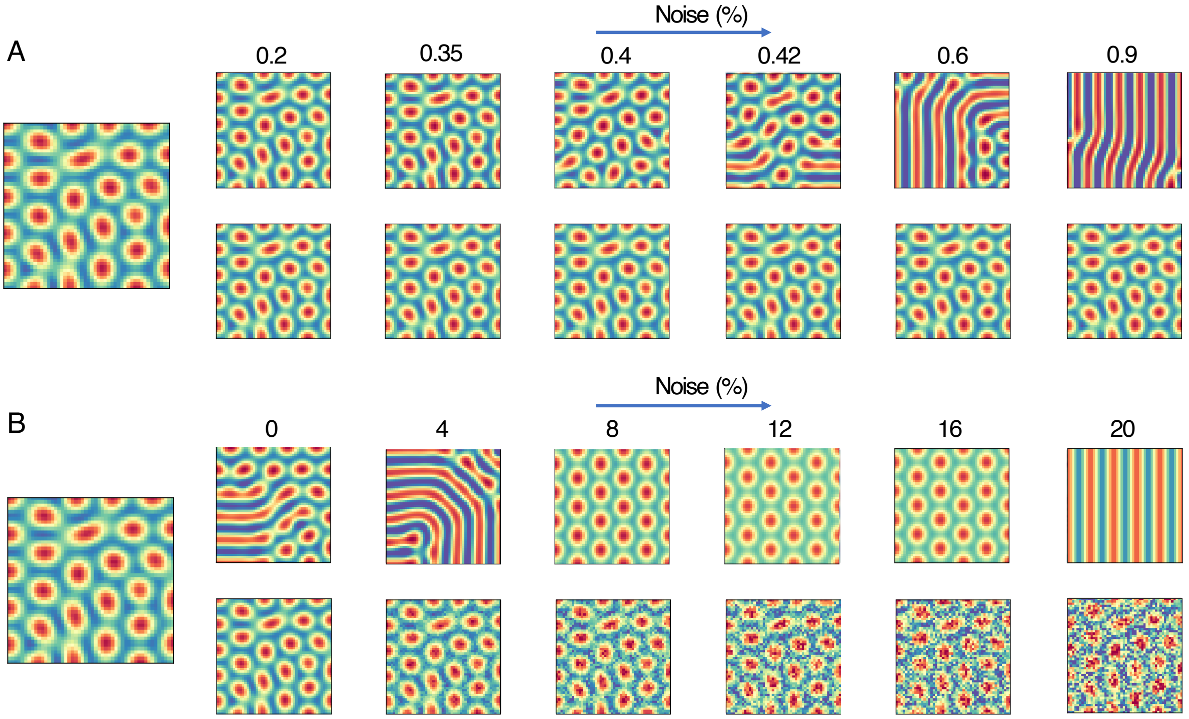

Fig. S1: Effect of noise in the recovered patterns for the Brusselator model. Related to STAR methods and Fig. 2C. In the top rows of both **(A)** and **(B)**, the resulting patterns obtained from LS (**A**) and RBF-PINNs (**B**) with different noise levels are shown. In the bottom rows the original pattern is corrupted by the respective level of noise shown, obtaining the input pattern to the methods. Note that, contrary to the examples in the main text, LS still produces parameters that output a pattern for high levels of noise, but the patterns shown go from the original dots to labyrinths and then to dots of different size. This is because as we increase the noise, the diffusion rates seem to approach zero, and do not escape from the Turing space, but instead change the type of patterns. If we increased the noise further, at around 20% noise with LS, we arrive at an extreme case of a dotted pattern with dots as small as pixels, similar to the 'salt and pepper' patterns. This is only the case for the LS method. For the RBF-PINN, we can see that the first two patterns obtained show spots with some labyrinths that seem to not have converged. Nevertheless, these are all converged patterns, and this show that small changes in the parameters take the original pattern to shift to labyrinths. As we increase the noise further, we arrive at a more stable dot pattern which nevertheless is still different from the original one, and at even larger noise we arrive at a labyrinth.

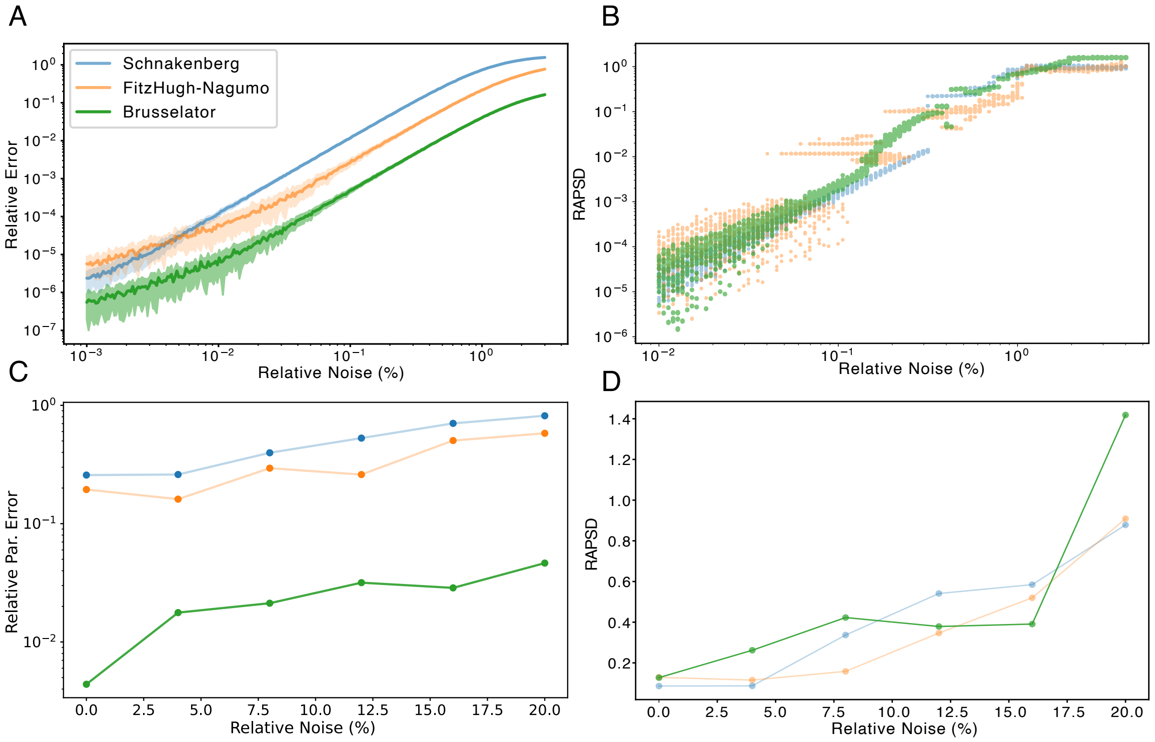

Fig. S2: Effect of noise in the relative error and RAPSD for the Brusselator model. Related to STAR methods and Fig. 2 D, E. **(A)** and **(C)** show the average relative error in parameters as a function of noise level applied to pattern before using LS and RBF-PINNs for the parameter inference, respectively. **(B)** and **(D)** show MSE between RAPS of original pattern and the ones obtained from LS and RBF-PINNs for different levels of noise, respectively. Note that the patterns obtained from RBF-PINN with 8 to 16% of noise are still very accurate results because, even though they might look different from the original pattern, they are very similar to the pattern in Fig. 1B in the main text, which was produced with the same parameter set but with a different initial condition. In the RAPS plot for the RBF-PINN we can observe an interesting behavior, the value without noise seems to have a higher RAPSD than the next four. This is not because the pattern is worst. Indeed, if we look at the pattern we can see that part of it matches the original one better than the rest. The problem is that the first pattern seems to have a part that shows labyrinths (it is shifting towards labyrinths) and another showing the same dots as the original, and the combination of the two is probably corrupting the RAPSD value.

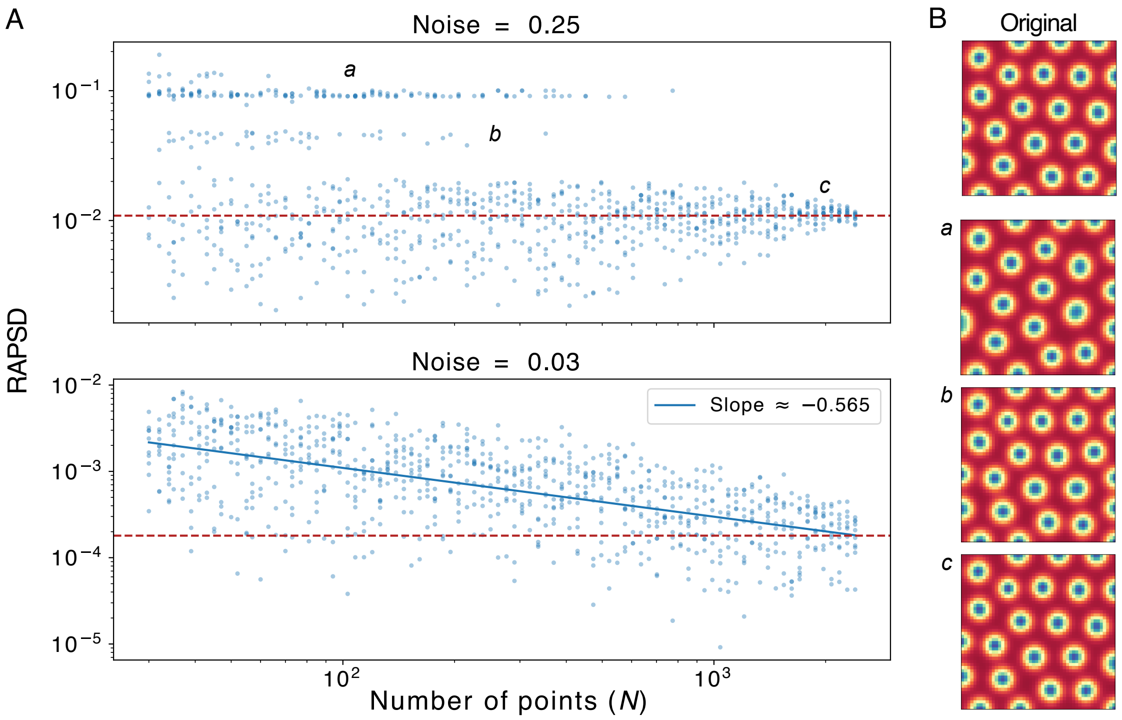

Fig. S3: Effect of the number of pixels on the RAPS difference. Related to STAR methods and Fig. 2. **(A)** RAPS difference measured from LS applied to different amounts of randomly selected points from a pattern that had been corrupted with two different levels of noise, 0.25 (top) and 0.03 (bottom). The dotted lines correspond to the RAPS difference value obtained when the whole pattern (all pixels) is used. These values can be seen in Fig. 2D, which shows the RAPS difference for the whole pattern as a function of noise. The solid line in the bottom plot shows the linear regression best fit. We can observe two different types of behaviors: (1) a monotonic improvement (bottom) when starting at a noise around 0.03, and (2) a step-wise improvement (top) when starting at a noise around 0.25. By looking at Fig. 2D, we can understand the different behaviors observed. A noise of 0.03 is in the zone of the plot where we observe a monotonic increase of RAPSD with increasing noise. Hence, we expect a similar result for decreasing the number of points, which is what we observe: the fewer points the more effect noise has. Similarly, as we increase noise from around 0.25, we have a step similar to the one observed in the top plot. Indeed, by comparing the RAPSD values, it can be seen that the region marked as (a) has a RAPSD value corresponding to the second step (around 0.1). In (**A**, top) we can also see an intermediate step (b), which is not observed in Fig. 2D, but this is because of the scale of the plot. It is worth pointing out that, unlike Fig. 2D, here we can see that the value of the RAPSD for a fixed N depends on the choice of points since in this case the noise is fixed (we sample points from the same corrupted pattern). This is expected since some points of the pattern will have a smaller noise, so depending on which ones are chosen the result will be more or less accurate. This stochasticity is more pronounced when the amount of points is smaller, which can be seen by the increase in spread of RAPSD as the number of chosen points is reduced. In the bottom plot we also show the line of best fit to the data, with a slope around -0.5, agreeing with the previously measured slope in Fig. 3C and the theoretical results mentioned in the main text. In **(B)** we show the different patterns for each of the regions in the top plot of **(A)**. The first one shows the original pattern, and the rest show regions (a), (b) and (c), respectively. It can be seen that the pattern at (c) looks visually the same as the original one, with imperceptible differences in position and scale of the dots. The pattern from (b) shows a slight difference in the right part of the plot, displaying an extra dot that does not appear in either (c) or the original, but with a similar wavelength. Finally, (a) shows a pattern with a completely different wavelength, which can be easily checked by counting the number of spots that appear on the pattern or their separation and width. All patterns have approximately the same scale, the one with the largest difference being (b).

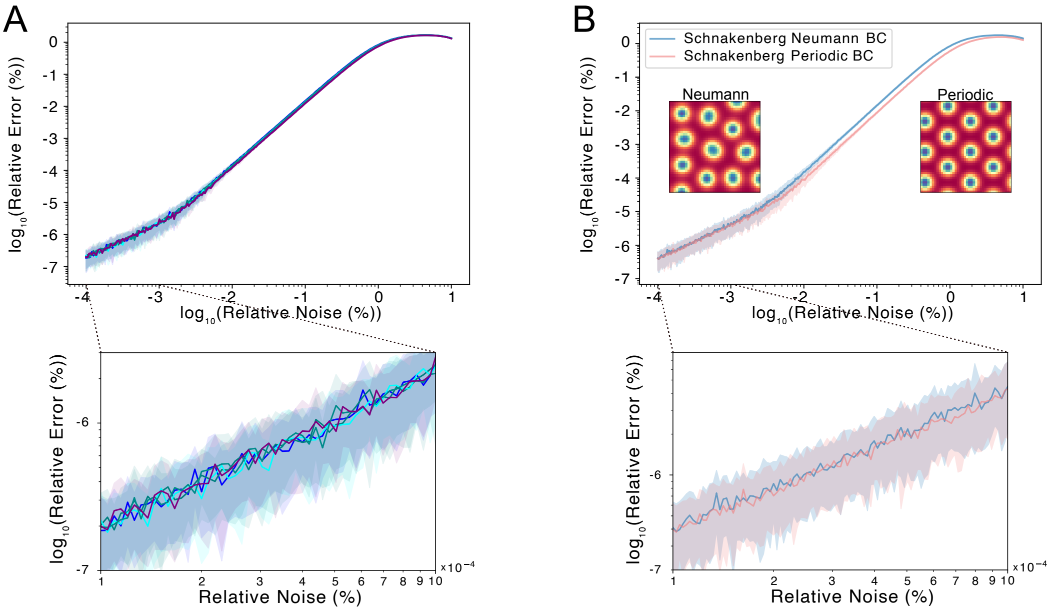

Fig. S4: Effect of initial (IC) and boundary (BC) conditions on LS. Related to STAR methods section *Initial and boundary conditions* and Fig. 2. **(A)** Plot of the mean and standard deviation of the relative error as a function of added noise for different ICs (performed with 60 repeats for the added noise at each value of the noise). Each of the five different ICs show the same trend, even though each one produces a slightly different pattern. **(B)** Plot of the mean and standard deviation of the relative error as a function of added noise for Neumann (blue) and periodic (orange) boundary conditions. The patterns used are shown inside the plot. For both plots, we provide a zoomed-in version around the small-noise region.

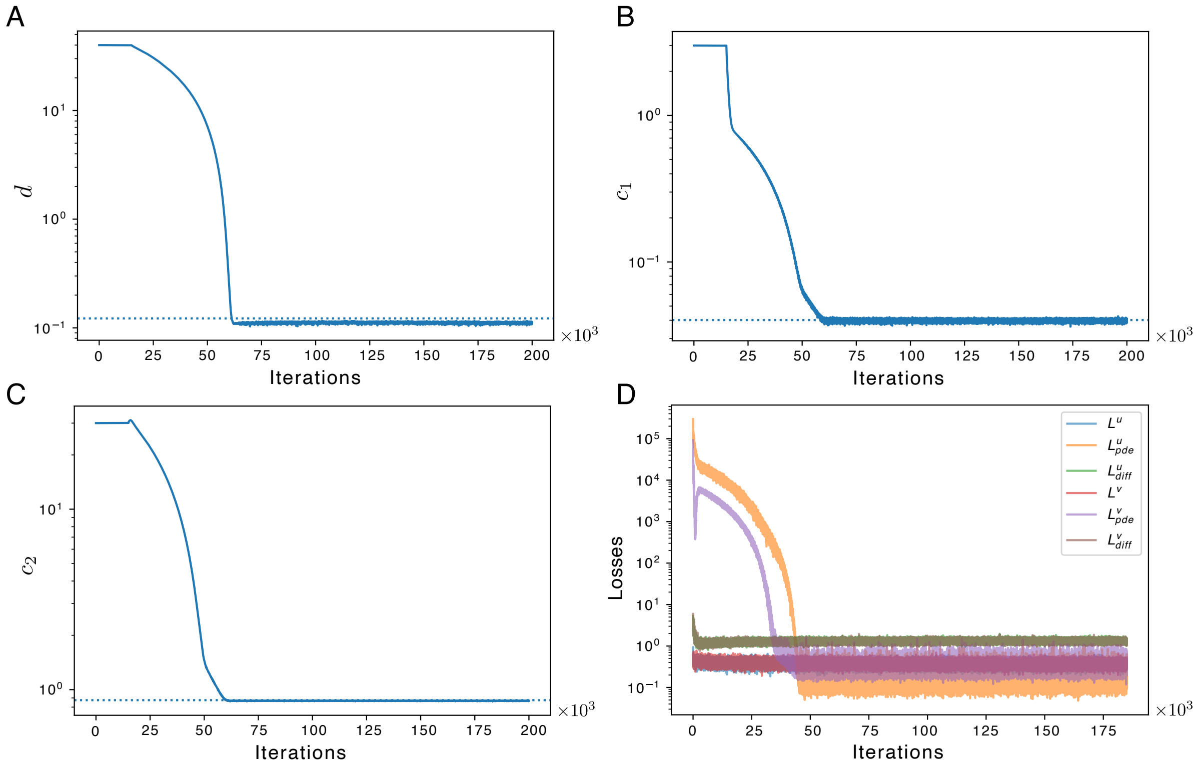

Fig. S5: Convergence plots of the parameters and the losses of the RBF-PINN. Related to STAR methods and Fig. 4. **(A)**, **(B)** and **(C)** Convergence of d, c_1, and c_2, respectively; the dotted lines represents the true parameters. **(D)** Convergence of the different losses of the network once the approximation phase of the training using RBFNN is finished. All plots correspond to the Brusselator model with a pattern corrupted by 20% noise.

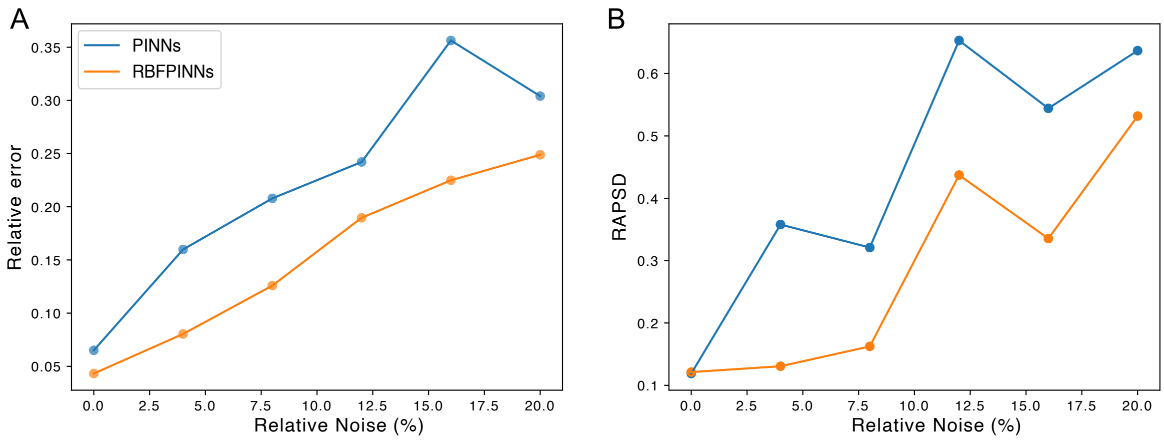

Fig. S6: Comparison RBF-PINNs with traditional PINNs. Related to STAR methods section *RBF-PINNs vs traditional PINNs* and Fig. 4. Plot of the average relative error in parameters **(A)** and the RAPSD **(B)** for PINNs (blue) and RBF-PINNs (orange) with the same losses and training time. We can see that for both measures RBF-PINNs performs better than traditional PINNs.

	Parameter List								
Model name	D_u	D_v	c_1	c_2	c_3	c_4	dx	Square size	Boundary Condition
Schnakenberg	1	40	0.1	1	1	0.9	1	50×50	Neumann
Schnakenberg	1	40	0.1	1	1	0.9	1	40×40	Periodic
Fitzhugh-Nagumo	0.05	0.00028	10	1	1	–	0.02	50×50	Neumann
Brusselator (spots)	0.0016	0.0131	4.5	8.72	–	–	0.02	50×50	Neumann
Brusselator (labyrinths)	0.0016	0.0131	4.5	6.96	–	–	0.02	50×50	Neumann

Table S1: **Model parameters and domain and boundary conditions specifications.** Related to STAR methods.
